# Ethnic differences in lens parameters measured by ocular biometry in a cataract surgery population

**DOI:** 10.1371/journal.pone.0179836

**Published:** 2017-06-27

**Authors:** Dajiang Wang, Behzad Amoozgar, Travis Porco, Zhen Wang, Shan C. Lin

**Affiliations:** 1 Department of Ophthalmology, University of California San Francisco, San Francisco, California, United States of America; 2 Department of Ophthalmology, General Hospital of People’s Liberation Army, Beijing, China; 3 Proctor Foundation, University of California San Francisco, San Francisco, California, United States of America; 4 Evidence-Based Practice Center, Mayo Clinic, Rochester, Minnesota, United States of America; 5 Mayo Clinic Robert D. and Patricia E. Kern Center for the Science of Health Care Delivery Mayo Clinic, Rochester, Minnesota, United States of America; 6 Division of Health Care Policy and Research, Department of Health Sciences Research, Mayo Clinic, Rochester, Minnesota, United States of America; National Eye Institute, UNITED STATES

## Abstract

**Purpose:**

To investigate whether differences exist in lens position and other lens parameters among major ethnic groups with cataractous eyes, which may help explain racial differences in angle closure risk.

**Methods:**

This retrospective, cross-sectional study included 807 adult patients who had cataract surgery between years 2014 and 2016 at the University of California, San Francisco (UCSF). Adult patients of white, Asian, Hispanic and African-American ethnicity were included. Lens position (LP), defined as anterior chamber depth (ACD) + 1/2 lens thickness (LT), was assessed using measurements from optical biometry. Other assessed biometric parameters included axial length (AL), relative lens position (RLP) (defined as LP/AL), and anterior chamber depth (ACD).

**Results:**

A total of 807 patients and 1361 eyes were included in this study from a database of patients having cataract surgery. Mean age was 69.2 years (age range from 18 to 101 years old), and 60.3% of patients were women. The mean LP measurements were 5.54±0.32 mm for white, 5.38±0.32 mm for Asian, 5.32±0.30 mm for Hispanic, and 5.40±0.28 mm for African-American participants. After adjusting for age, sex, and AL, significant differences were found when comparing LP in paired comparisons among White cohort with Asians (*P*<0.001), Hispanics (*P*<0.001) and African-Americans (*P* = 0.003). Additionally, when comparing RLP, similar significant results were found when comparing Whites with Asians (P<0.001), Hispanics (*P<*0.001) and African-Americans (*P* = 0.002). Lastly, pair-wise comparison of LT between ethnic groups showed significant differences while comparing Asians with Whites (*P* = 0.001) and Asians with African-Americans (*P*<0.001).

**Conclusion:**

The results of this study suggest that the LP of Hispanic, Asian, and African-American patients are significantly smaller than that of White patients, and among all ethnic groups, Hispanics and after Asians have the smallest LP (*P*<0.001) and RLP (*P*<0.001). These findings may have implications for the relative risk of angle closure and the potential IOP response after cataract surgery among different ethnic groups.

## Introduction

Glaucoma is a major public health challenge, being the leading cause of irreversible blindness worldwide. It has been estimated that 60.5 million people were affected by primary open-angle glaucoma (POAG) and primary angle-closure glaucoma (PACG) globally in 2010.[1–4]

Elevated intraocular pressure (IOP) is the most important modifiable risk factor associated with glaucoma development.[5] Many experimental studies have demonstrated sustained intraocular pressure reduction after routine cataract extraction in eyes with or without ocular hypertension or glaucomatous disease.[6–8] So far, the mechanism remains poorly understood and the magnitude of this effect is highly variable and unpredictable. In patients with narrow angles, the IOP-lowering effect appears to also be proportional to the degree of anterior chamber angle deepening induced by cataract surgery.[9] However, for POAG, the mechanism is not as clear.[10,11] Many studies have focused on the relationship between the ocular anatomy and IOP reduction after phacoemulsification. Anterior chamber and angle parameters have been found to be predictive factors. For example, the ratio of the preoperative IOP and anterior chamber depth (ACD)—the pressure-to-depth ratio (PD ratio)—and changes in angle opening distance (AOD) have been reported to be associated with IOP reduction after phacoemulsification in non-glaucoma subjects.[9,12]

Lens position (LP)—defined as LP = ACD + 1/2 lens thickness (LT)—and relative lens position (RLP)—defined as RLP = LP/axial length are more easily computed from measurements obtained through optical ocular biometry, which is part of routine testing for intraocular lens (IOL) power calculations prior to cataract surgery. They could also be used to understand how the lens affects the IOP reduction seen in previous studies. Our former research found that the percentage of IOP reduction after cataract surgery in non-glaucomatous eyes with open angles is greater in patients with more anteriorly positioned lenses.[13] In addition, we have also shown that LP is an accessible predictor with considerable predictive value for postoperative IOP change.[14]

Another area in which LP may be helpful is the understanding of the risk factors and possible treatment for PACG. Progressive shallowing of the anterior chamber (AC) in predisposed eyes is mostly attributable to age-related increase in lens thickness and more anterior positioning of the lens.[15] The restoration of a deeper angle configuration by removing a thickened and anteriorly positioned lens may be advantageous in eyes with PACG and may lead to a significant IOP reduction.[16,17] Furthermore, differences in ocular anatomy may contribute to ethnic differences in glaucoma risk, particularly for PACG. Previous studies showed that a more anterior lens is related to higher risk for angle closure.[18–20]

In this study, we compare the LP and other lens parameters among White, Asian, Hispanic and African-American subjects. We hypothesize that LP is significantly different among ethnicities that are at different risk for developing PACG and that they may respond differently in terms of IOP change after cataract surgery.

## Methods

### Study design

This retrospective, cross-sectional study was approved by the University of California, San Francisco (UCSF) Committee on Human Research, and the study adhered to the tenets of the Declaration of Helsinki. Due to the retrospective nature of the study, and since the data were analyzed anonymously, UCSF Committee on Human Research determined that it was not necessary to obtain the participants' consent. This study enrolled consecutive subjects who met the inclusion criteria and underwent cataract surgery at the UCSF general ophthalmology and subspecialty clinics between January 1, 2014, and January 31, 2016.

The ethnicities of study participants included were White, Asian, Hispanic and African-American. Ethnicity was assessed by self-report. The Asian cohort included individuals of self-reported Chinese, Japanese, Korean, Filipino, and Vietnamese descent. The White cohort included only those of European-derived ancestry. **Inclusion criteria** included: 1) adult patients (18 years or older); 2) self-reported White, Asian, African-American, or Hispanic ethnicity; and 3) optical biometry with the Lenstar (model LS 900, Haag-Streit AG, Koeniz, Switzerland) prior to planned cataract surgery. **Exclusion criteria** included

1) self-reported biracial ancestry; 2) uveitis, severe retinal disease such as wet macular degeneration, or congenital anomalies; 3) history of ocular trauma or any prior intraocular surgery; 4) history of intraocular laser treatment; 5) use of steroid or glaucoma drops within the 3 months prior to optical biometry; 6) contact lens use; or 7) inability to conduct the necessary testing. Both eyes of the patients with cataracts were included in the study if they met the inclusion and exclusion criteria. However, not all the eyes underwent cataract surgery after the measurement. In reviewing the medical charts, we ensured that all eyes included in the study were eligible based on inclusion and exclusion criteria. Mixed effects regression modeling was used to adjust for the use of both eyes in some subjects.

### Data collection

The study participants underwent ophthalmologic examinations, including visual acuity assessment, refractometry, keratometry, intraocular pressure measurement by Goldman applanation tonometry, slit-lamp biomicroscopy, and optical biometry which provided data on axial length (AL), anterior chamber depth (ACD), lens thickness (LT), and central corneal thickness (CCT). We conducted optical biometry with the Lenstar. Five readings were taken for each eye, and after omitting the highest and lowest values, the mean of the remaining three readings was used for analysis. All measurements were done for both eyes of all subjects. All enrollees received an ophthalmic examination that included refraction. Trained ophthalmic technicians (R.I.C. and D.T.B.) performed all scans.

Lens position (LP) was calculated as LP = ACD + 1/2 (LT), and relative lens position (RLP) was defined as RLP = LP/AL.

### Statistical analysis

We used descriptive analyses for the demographic data related to the ethnic cohorts. Nonparametric Wilcoxon rank-sum tests were used to compare cohorts regarding LP, RLP, CCT, LT, and AL. We used linear mixed-effect models to compare differences in LP and RLP between ethnic cohorts while accounting for each eye as a separate entity to maximize the effect of randomization plus including both eyes for analysis. Two-tailed *P* value <0.05 was deemed statistically significant. All statistical analyses were conducted using Stata version 14.0 (Stata Corp LLP, College Station, TX, USA).

## Results

Over the study period, there were a total of 2173 eyes assessed using Lenstar optical biometry. Four hundred and fifty-six eyes were excluded because of missing ethnicity information, self-reported biracial ancestry, or ethnicity other than the 4 major groups included in this study; 246 were excluded because of their history of glaucoma; and 110 were excluded because of history of surgery or other eye diseases. After these exclusions, a total of 807 patients and 1361 eyes were included in the study. There were 729 White, 386 Asian, 141 Hispanic and 105 African-American eyes. There were 60.3% women and the mean age of the sample was 69.2 ± 12.7 years. Distribution of laterality was 49.7% right eyes.

Among the entire study group, the mean CCT was 543.81 ± 36.18μm, the mean ACD was 3.19 ± 0.43 mm, the mean LT was 4.54 ± 0.47 mm, and the mean AL was 24.16 ± 1.64 mm. The mean IOP was 16.43 ± 3.07 mmHg.

Table 1 shows the comparison of the demographic and clinical characteristics of the ethnic cohorts. The mean LP measurements were 5.54 ± 0.32 mm for Whites (largest), 5.38 ± 0.32 mm for Asians, 5.32 ± 0.30 mm for Hispanics (smallest) and 5.40 ± 0.28 mm for African-Americans. The mean RLP measurements were 0.230 ± 0.010 for whites, 0.222 ± 0.010 for Asians, 0.224 ± 0.010 for Hispanics and 0.224 ± 0.010 for African-Americans.

**Table 1:** Comparison of demographic and clinical characteristics of the ethnic cohorts.

Characteristic	White	Asian	Hispanic	African American
Participants, No.	442	223	82	60
Eyes, No.	729	386	141	105
Age, mean (SD), y	67.6 (12.4)	72.07(11.32)	67.70(15.23)	67.22(13.33)
Female sex, No. (%)	245(55.30%)	146(65.47%)	45(54.88%)	42(70.00%)
CCT, mean (SD), mm	550.61(34.86)	539.24(33.15)	539.79(36.25)	518.81(41.49)
ACD, mean (SD), mm	3.28(0.42)	3.08(0.43)	3.06(0.39)	3.16(0.43)
LT, mean (SD), mm	4.52(0.46)	4.60(0.46)	4.53(0.44)	4.48(0.54)
Al, mean (SD), mm	24.26(1.56)	24.23(1.86)	23.59(1.50)	24.05(1.30)
IOP, mean (SD),mmHg	16.32(2.99)	16.46(3.01)	16.64(3.01)	16.91(3.73)
LP mean (SD), mm RLP mean (SD), mm	5.54(0.32) 0.23(0.01)	5.38(032) 0.222(0.01)	5.32(0.30) 0.224(0.01)	5.40(0.28) 0.224(0.01)

CCT = central corneal thickness, ACD = anterior chamber depth, LT = lens thickness. AL = axial length, IOP = intraocular pressure, LP = lens position, RLP = relative lens position

The univariate linear analysis showed that age, sex and AL were potential confounders with LP. The differences between each ethnicity's LP and RLP and those of the reference group (White) are depicted in Tables 2 and 3, respectively. Using a multivariable linear mixed-effect regression model with Whites as the comparator, adjusted for age, sex and AL, we found that significant differences exist compared to the LP in the Asian group (β coefficient = -0.14, 95% CI, -0.18 to -0.09, *P*<0.001), African-American group (β coefficient = -0.11, 95% CI, -0.18 to -0.04, *P* = 0.003) and Hispanic group (β coefficient = -0.16, 95% CI, -0.22 to -0.10, *P*<0.001). Thus, all other races had smaller LP than Whites. The biggest differences were seen in Hispanics and Asians compared to Whites (*P*<0.001).

**Table 2:** Comparisons of lens position (LP) between ethnic cohorts.

LP	coefficient	Std. Err	95% low CI	High CI	*P* value
White	reference				
Asian	-0.14	0.02	-0.18	-0.09	<0.001
Hispanic	-0.16	0.03	-0.22	-0.10	<0.001
African American	-0.11	0.04	-0.18	-0.04	0.003

*P* values by multivariable, linear mixed-effect regression models, adjusted for age, sex, and axial length.

**Table 3:** Comparisons of relative lens position between ethnic cohorts.

RLP	coefficient	Std. Err	95% low CI	High CI	P value
White	reference				
Asian	-0.55 x10^-2^	0.09 x10^-2^	-0.72 x10^-2^	-0.37 x10^-2^	<0.001
Hispanic	-0.62 x10^-2^	0.13 x10^-2^	-0.88 x10^-2^	-0.36 x10^-2^	<0.001
African American	-0.46 x10^-2^	0.15 x10^-2^	-0.76 x10^-2^	-0.16 x10^-2^	0.002

*P* values by multivariable, linear mixed-effect regression models, adjusted for age sex and AL

Comparison of RLP using multivariable linear mixed-effect regression model, adjusted for age, sex and AL, using White as the reference group, showed that a significant different of RLP exists between ethnic cohorts. The highest negative effects (estimated difference) were seen in Hispanics (coefficient = -0.0062, 95% CI, -0.88 x10^-2^ to -0.36 x10^-2^, *P*<0.01) and after that in Asians (coefficient -0.0055, 95% CI, -0.72 x10^-2^ to -0.37 x10^-2^, *P*<0.01) as compared to White and the lowest negative effect (estimated difference) was seen in African-Americans as compared to White (coefficient = -0.0046, 95% CI, -0.76 x10^-2^ to -0.16 x10^-2^, *P* = 0.002).

Running our multivariable linear mixed-effects regression model test, comparing LP and RLP but using Asians as the baseline, we found significant differences when comparing Asians to African-Americans, White and Hispanic. As a result, we found the lowest values (highest negative effect) of LP and RLP in Hispanics and after that group in Asians while comparing the ethnic cohorts.

Furthermore, pairwise comparison of LT showed that Asians significantly differed from both African-Americans (*P*<0.001) and Whites (*P* = 0.001) but not Hispanics (Table 4). We also found that Asians had the thickest LT and African-Americans had the thinnest (Table 1).

**Table 4:** Pair-wise comparison of lens thickness between ethnicities (p values are presented).

	White	Asian	Hispanic	African American
White		0.001	0.56	0.19
Asian			0.08	<0.001
Hispanic				0.13
African American				

## Discussion

In the present study of a convenience sample of patients scheduled to have cataract surgery, using linear mixed-effect regression models, we found Hispanics have the smallest LP and RLP and Asians have the second smallest LP and RLP while having the greatest lens thickness (LT) value (Table 1). The LP of Whites was greater than all other racial groups (P<0.05 for all comparisons) and the RLP was greater than other races as well. Our findings may help provide anatomic insight into the relative risk for PACG among different ethnic groups and the potential efficacy of cataract surgery on IOP in these different groups.

Angle-closure glaucoma is an anatomical disorder of the anterior segment of the eye characterized by permanent closure of part of the filtration angle. Demographic and anatomical factors play significant roles in the development of angle-closure glaucoma.[21–23] Ethnicity was recognized as a major risk factor for primary angle-closure glaucoma in a comprehensive review of the literature conducted by Congdon et al in 1992, with Asians among those with the highest risk and Whites having substantially lower risk.[24] Previous studies have also shown that greater lens thickness (LT), smaller anterior chamber depth (ACD), and shorter axial length (AL) are risk factors for PACG.[25–29] Recent studies have also shown that LP, RLP, and LT can be predictive for angle closure.[30–32] In this current study of a clinic population, our findings suggest that the position and thickness of a lens may also be significantly different among ethnic groups and help explain the differential risk for angle closure among these groups. Hispanics and Asians had the smallest LP and RLP and the thickest LT among the four major racial groups in our study.

Asians have the highest prevalence of PACG.[33–35] Furthermore, a population-based study by Quigley et al. reported that 0.1% of their Hispanic population were affected with PACG.[36] However, it has been speculated that angle closure might be more common among Hispanic individuals in the Western Hemisphere because of their linked heritage to migrants from Asia during the last Ice Age.[36] Sakata K et al. found that PACG was more common among south Brazilians (Hispanic) than in European populations, and the adjusted prevalence of PACG observed in the study suggested that the prevalence of this disease in non-Asian, such as Hispanic, populations may be greater than has been traditionally believed and that most cases are asymptomatic.[37] Interestingly, in our study, the highest LP was recorded in the White group and the lowest measurement was recorded in the Hispanic group. In Whites, these findings are consistent with the low rate of PACG in this group.

Recent studies investigated different biomechanical parameters such as lens vault, trabecular meshwork height and iris thickness between different racial groups.[38–41]

However, there have not been any prior studies describing the differences in LP, RLP, and LT among the major major racial groups. Differences in lens position, thickness, and anterior chamber depth in different racial groups and the continued growth of the lens throughout life[42] may be partially responsible for the dissimilarity in the presenting demographic features of patients with PACG.

In 1970, Lowe et al. found that the eyes with chronic ACG are smaller than normal eyes, and the lens is situated relatively more anteriorly in the eye when compared with the lens position in matched normal eyes.[15] We also found that there is a significant relationship between LP and age (and thus, we included the age in the mixed effects regression model as confounder), which means the lens is closer to the cornea as age increases. This substantiates findings in previous studies.[30] According to the iris-lens canal theory, the more anteriorly the lens is positioned, the more likely it is to result in “partial pupillary block.”[43] The posterior chamber–anterior chamber pressure gradient is inversely proportional to the height of the iris-lens canal. When the lens is more anteriorly positioned and the height is decreased, the higher pressure gradient will cause a situation similar to pupillary block.[44]

In our previous study, we found that LP was an accessible parameter with considerable predictive value for postoperative IOP change after cataract surgery in non-glaucomatous patients with open angles.[14] We also found that there is a trend towards a similar effect in POAG eyes.[45] The results of the present study therefore suggest that Asians and Hispanics may benefit more from cataract surgery in terms of IOP control.

Additionally, in our data, the highest LT—another risk factor for PACG[26,46]—was in the Asian group. In patients with PACG, the anterior segment is more crowded because of the presence of a thicker, more anteriorly located lens.

**Fig 1:**
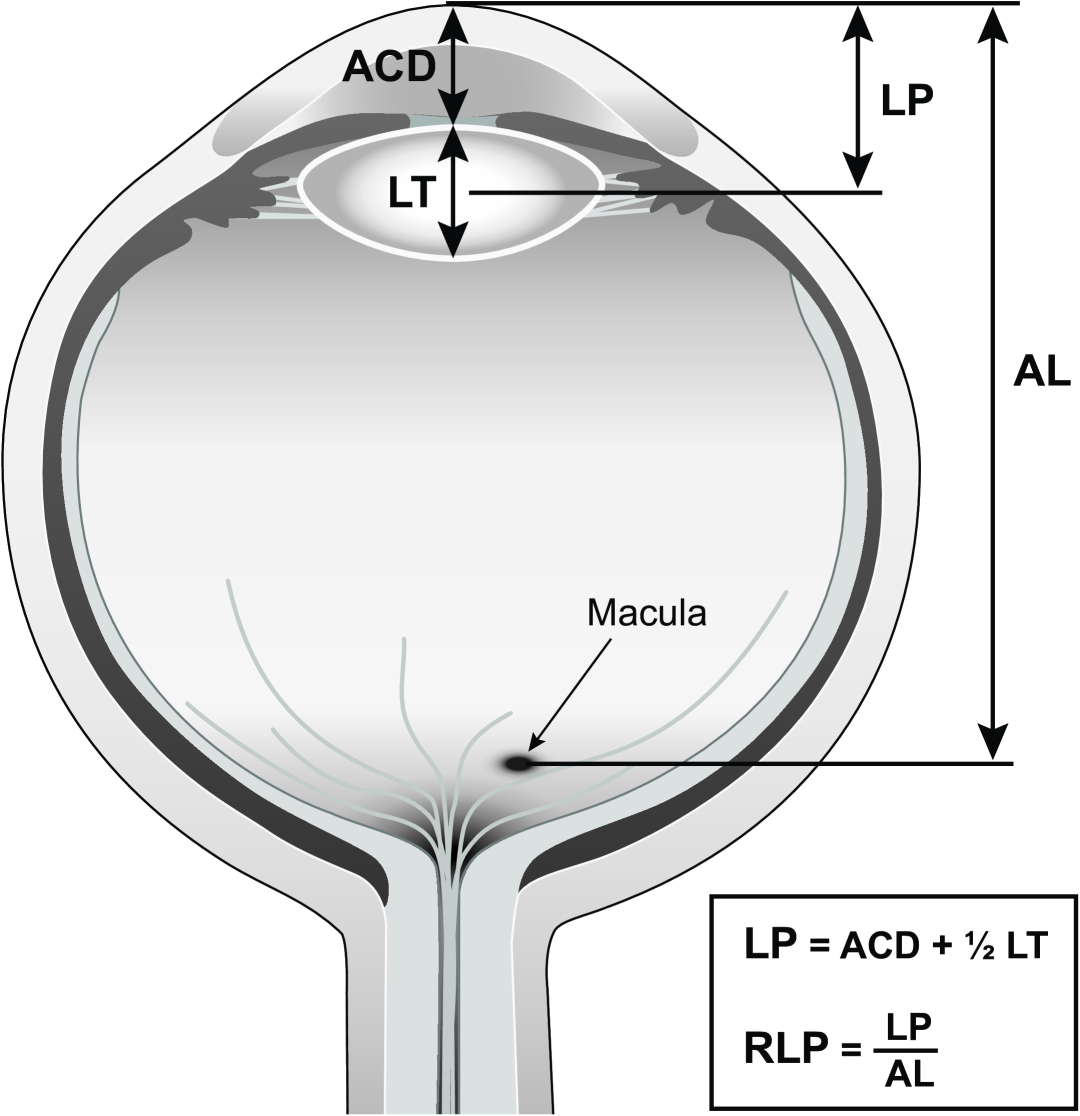
Illustrates lens parameters based on Lenstar measurements.

One prominent advantage of this study was the use of the Lenstar LS 900 for ocular biometry, a device which has been shown to have high accuracy and can measure CCT, ACD, LT and AL, in addition to keratometric (K) readings and corneal diameter (CD). This machine measures AL from the surface of the cornea (epithelium including the tear film) to the macular pigmentary epithelium and ACD from the surface of the cornea to the anterior surface of the crystalline lens (Fig 1). Shammas et al. reported that the precision of the measurements obtained by the Lenstar was extremely high.[47]

Although the same precision cannot be applied regarding the interchangeability of measurements between AS-OCT, IOLMaster and Lenstar, the reproducibility of measurements (precision) within the Lenstar device is excellent.[48]

The primary limitation of this study is the use of retrospective data from a convenience sample of subjects scheduled to have cataract surgery at UCSF. However, this feature should not necessarily bias the results in any particular direction such that certain racial groups would have greater likelihood of smaller or greater LP, RLP, and LT. Furthermore, we adjusted for potential confounding factors such as age, gender, and axial length when appropriate. Additional limitations of this study include the relatively small sample size in some groups, investigating lens parameters among cataract surgery population, and the self-reported nature for ethnicity in our study. Moreover, each ethnic group included in this study also has substantial intragroup differences. Self-identification within an ethnic group also does not preclude genetic contribution from another ethnic group. To minimize intra-ethnic variation, we excluded individuals of biracial ancestry. However, it is possible that there is still great genetic diversity within each cohort. Furthermore, this is a retrospective study and parameters from procedures such as gonioscopy and ASOCT were not consistently available. Although IOP is available, we did not evaluate this factor as an outcome because it was assessed by different technicians and devices and at various times of the day, without a fixed protocol.

In summary, we found significant differences in the lens position and relative lens position among various ethnic groups, with Whites having the greatest LP and RLP. In addition, Hispanics followed by Asians had the smallest LP and RLP. These findings may have relevance as to the relative risk for ACG in these groups and potentially in the IOP outcome after cataract surgery. Our results can serve as a starting point for designing future prospective observational studies which have larger populations and a variety of ethnicities.
